# Urinary Metabolomic Study of Chlorogenic Acid in a Rat Model of Chronic Sleep Deprivation Using Gas Chromatography-Mass Spectrometry

**DOI:** 10.1155/2018/1361402

**Published:** 2018-02-11

**Authors:** Wei-ni Ma, Ming-mei Zhou, Xiao-jun Gou, Le Zhao, Fang Cen, Ying Xu, Hong-yi Shen

**Affiliations:** ^1^Center for Chinese Medicine Therapy and Systems Biology, Shanghai University of Traditional Chinese Medicine, Shanghai 201203, China; ^2^Central Laboratory, Baoshan District Hospital of Integrated Traditional Chinese and Western Medicine of Shanghai, Shanghai University of Traditional Chinese Medicine, Shanghai 201999, China; ^3^Experiment Center of Teaching & Learning, Shanghai University of Traditional Chinese Medicine, Shanghai 201203, China; ^4^Department of Physiology, Shanghai University of Traditional Chinese Medicine, Shanghai 201203, China; ^5^Research Center for Health and Nutrition, Shanghai University of Traditional Chinese Medicine, Shanghai 201203, China

## Abstract

The urinary metabolomic study based on gas chromatography-mass spectrometry (GC-MS) had been developed to investigate the possible antidepressant mechanism of chlorogenic acid (CGA) in a rat model of sleep deprivation (SD). According to pattern recognition analysis, there was a clear separation among big platform group (BP), sleep deprivation group (SD), and the CGA (model + CGA), and CGA group was much closer to the BP group by showing a tendency of recovering towards BP group. Thirty-six significantly changed metabolites related to antidepressant by CGA were identified and used to explore the potential mechanism. Combined with the result of the classic behavioral tests and biochemical indices, CGA has significant antidepressant effects in a rat model of SD, suggesting that the mechanism of action of CGA might be involved in regulating the abnormal pathway of nicotinate and nicotinamide metabolism; glyoxylate and dicarboxylate metabolism; glycine, serine, and threonine metabolism; and arginine and proline metabolism. Our results also show that metabolomics analysis based on GC-MS is a useful tool for exploring biomarkers involved in depression and elucidating the potential therapeutic mechanisms of Chinese medicine.

## 1. Introduction

With the accelerated pace of modern society life and the rapid increase of the working pressure, more and more people are sleep deprived for various reasons, such as extending working hours into the night and curtailing or delaying sleep. Accumulating studies have demonstrated that sleep loss is closely coupled with an increased risk of immune system imbalance [1], neuroendocrine dysfunction [2], and depression [3, 4]. Depression and sleep disturbance are closely linked and have a mutual cause-and-effect relationship. Depressive patients usually suffer from poor sleep quality, while sleep disorder has also become a diagnostic criterion for major depressive disorder (MDD) [5]. However, the mechanism by which sleep loss causes depression is still unclear. At present, most of the drugs for the treatment of depression are mainly western medicine in clinical work, but, there are many problems such as recurrence and toxic side effects after drug withdrawal, which greatly affects the clinical efficacy. Chinese medicine has the characteristics of stable curative effect, lasting effect, and little adverse reaction. It has a long history in the prevention and treatment of depression and has become a hot topic both at home and abroad [6]. Recent studies demonstrate that polyphenols from natural herbs have the feasibility to be potential therapeutic agents in promoting resilience against SD-induced dysfunction [7, 8]. Chlorogenic acid (CGA), one of the major polyphenols of many fruits, has been reported in a number of previous studies as an antioxidant and neuroprotectant compound [9, 10]. CGA is also enriched in many traditional Chinese herbs, such as the bark of *Eucommia ulmoides* Oliver, leaves of *Hypericum origanifolium*, flowers of *Lonicera japonica*, fruits of *Crataegus oxyacantha* Linn., and whole herb of *Artemisia capillaris* Thunb. [11]. Studies showed that CGA could exhibit an antidepressive effect as shown in animal behavioral tests, accompanied by neuron protection and promotion of serotonin release in the brain [11, 12]. In addition, the evidence that decaffeinated coffee enriched in CGA elevated mood in 39 healthy volunteers also demonstrated CGA's character as an antidepressant/mood-elevator agent. However, the studies to address CGA's efficacy as well as the mechanism in treating depression are not sufficient [13].

As one of the hypothesis-free approaches, metabolomics is a powerful tool in discovering novel molecules involved in the pathophysiological process of various diseases [14]. Our previous metabolomic studies have revealed significant changes of systemic metabolites in novel depression rat model [15, 16]. Therefore, in order to develop pathophysiological understanding of the underlying relationship between SD stress and depression [17, 18], a metabolic method based on GC-MS with multivariate statistical techniques was used to assess the efficacy of CGA in a rat model of sleep deprivation in this study. In addition, potential biomarkers involved in antidepressant effects were identified and explored the underlying mechanism.

## 2. Methods

### 2.1. Rats and Treatments

Eighteen healthy male Wistar rats (purchased from Shanghai Sippr-BK Laboratory Animal Co. Ltd.) weighing 230–250 g were kept at a density of 6 per cage. All rats were maintained in a standard laboratory environment (20 ± 5°C and 55 ± 15% humidity) under a 12 h dark/light cycle (07:00–19:00 at 40 W light condition) with free access to a semipurified diet [19] and water ad libitum for 1 week prior to the experiment. The rats were randomly divided into three groups (*n* = 6): (1) big platform group (BP), (2) small platform group also known as sleep deprivation group (SD), and (3) CGA-treated group (SD + CGA); animals received CGA (purity ≥ 98%, HPLC method, Sichuan Weikeqi Bio-Tech Co. Ltd., China) extracted from *Eucommia ulmoides* Oliver bark and dissolved in anhydrous ethanol as a published paper performed [12] at a daily dose of 50 mg/kg of body weight, orally, while BP and SD group received normal saline solution from day 1 to day 7. Sleep deprivation procedure was carried out as previously reported [18]. During the study period, the experimental room was maintained at controlled conditions (20 ± 5°C and 55 ± 15% humidity) under a 12 h dark/light cycle (07:00–19:00 at 40 W light condition). The BP group was kept in the same environment with wide platforms (16 cm in diameter). Food and water were provided ad libitum by hanging on top of the tank. The research was approved by the Ethics Committee of the Shanghai University of TCM. Animal welfare and experimental protocols were strictly in accordance with the Guide for the Care and Use of Laboratory Animals and the ethics and regulations of the Shanghai University of TCM.

### 2.2. Behavioral Test

At the end of the SD paradigm, rats were subjected to FST and TST as previously performed [12] with little modification. Briefly, in the FST, rats were placed in a glass cylinder (19 cm in diameter and 50 cm in height) filled with 23–25°C water (30 cm in depth) for 6 min, individually. Immobility was defined as rats hopelessly kept heads above the water without other motions. In the TST, rats were suspended 50 cm above the floor by fixing the tip of the tail (1 cm). The total test procedure was counted during a test period of 5 min. Rats were considered immobile only when they hung passively in the absence of all movements.

### 2.3. Sample Collection

Each rat was put into a metabolic cage for 24 h urine sample collection after SD paradigm. Urine was centrifuged at 4°C, 13,000 rpm for 15 min, and the supernatant was stored at −80°C prior to GC-MS analysis. Subsequently, rats were sacrificed with chloral hydrate (300 mg/kg body weight, i.p.) anaesthesia for collection of abdominal aorta blood, which was centrifuged at 13,000 rpm and 4°C for 15 min, and the serum was collected and stored at −80°C.

### 2.4. Biochemical Analysis

Plasma concentrations of IL-6, TNF-*α*, CORT, and NE were determined by an automatic biochemistry analyzer using the commercial ELISA kits according to the protocols provided by the manufacturer.

### 2.5. Urine Sample Preparation, Derivation, and GC-MS Analysis

Prior to analysis, urine samples were thawed at room temperature and vortex-mixed for 30 s. Pretreatment procedure was in accordance with our previous published method [20] with little modification. Briefly, each 200 *μ*L aliquot of urine samples was added into a 1.5 mL tube for centrifugation (13,000 rpm, 4°C for 10 min). Later, a 50 *μ*L supernatant was transferred to a new tube containing 10 *μ*L urease (30 U) and incubated at 37°C for 15 min. Then the metabolite extraction procedure was carried out after adding two internal standard solutions (10 *μ*L of L-2-chlorophenylalanine in water, 0.3 mg/mL; 10 *μ*L of heptadecanoic acid in methanol, 1 mg/mL) and 170 *μ*L of methanol. After vortexing for 30 s, the mixture was centrifuged at 13,000 rpm (4°C) for 5 min. A 200 *μ*L supernatant was transferred into GC vial and dried under in a stream of pure nitrogen gas at 30°C. The two-step derivatization process was the same as Qiu et al.'s method [21].

Each 1 *μ*L derivatized sample was injected into the GC-MS instrument Agilent 6890/5975B GC/MSD system (Agilent Technologies, California, USA) in splitless injection mode. A programmed column temperature was optimized for successful separation (Table 1). Ion separation was achieved on a capillary column (Agilent J&W DB-5ms Ultra Inert 30 m × 250 *μ*m, i.d., 0.25 *μ*m film thickness) with high-purity carrier gas (helium) at a constant flow rate of 1.0 mL·min^−1^. The temperatures of the injection port, transfer interface, and ion source were set to 280°C, 260°C, and 230°C, respectively. The measurements were collected using electron impact ionization (70 eV) in full scan mode (*m*/*z* 50–600).

### 2.6. Data Analysis

Data from the behavioral test and biochemical detection were analyzed using SPSS statistical package (SPSS program, version 21.0). Statistical analysis was performed using one-way ANOVA followed by LSD *t*-test. The results were expressed as means ± standard errors of the mean (SEM). Significant differences were considered at a level of *P* < 0.05 or *P* < 0.01. The graphs were generated using Prism 6.0 (GraphPad Software Inc., USA).

All the GC-MS raw files (.D) of urine samples were converted into easily identified NetCDF format via Agilent MSD workstation and subsequently preprocessed using the XCMS toolbox script with R 2.13.2 package with default settings for baseline correction, peak recognition, and calibration. The resulting data were exported into Microsoft Excel, and the peaks were normalized to the total sum of spectrum. The resulting three-dimensional matrix involving peak index (RT-*m*/*z* pair), sample numbers (observations), and normalized peak area percent were introduced into SIMCA-P 11.0 software package (Umetrics, Umeå, Sweden) for multivariate analyses including principal components analysis (PCA) and orthogonal partial least squares discriminant analysis (OPLS-DA). The quality of the constructed model was evaluated by R2X, R2Y, and Q2 parameters. The former two were used to assess the goodness of fit, and the last one was used to assess the predictive reliability of the model [22].

### 2.7. Metabolite Identification and Pathway Analysis

Metabolite identification was performed firstly with an already constructed standard library including retention time and mass spectra. The peaks which could not match with standard library were imported to the NIST MS 2.0 (NIST, Gaithersburg, MD) software to search compound information from the NIST 11 library. Compounds with a similarity of more than 70% were finally verified as available reference compounds. Online resource HMDB (http://www.hmdb.ca/) was applied to confirm endogenous metabolites from the above-identified compounds. The MetaboAnalyst 3.0 was used to forecast metabolic pathways significantly affected by those metabolites.

## 3. Results

### 3.1. CGA Intervention Reversed Depressive Behaviors Induced by SD

**Figure 1:**
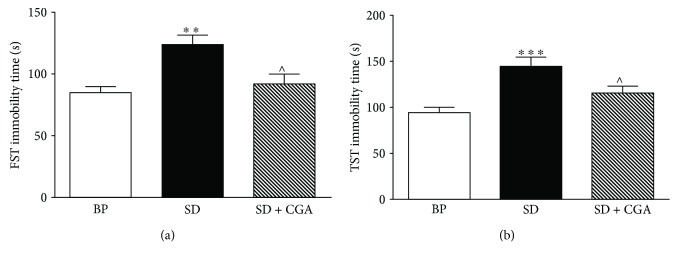
Behavioral test results of three groups (BP: big platform group, SD: sleep deprivation group, SD + CGA: SD group with CGA intervention). Data are expressed as mean ± SEM (*n* = 6). Compared with the BP group, ^∗∗∗^*P* < 0.001; compared with the control group, ^∗∗^*P* < 0.01; and compared with SD group, ^∧^*P* < 0.05.

As shown in Figure 1, the immobility time of rats in the FST and TST was increased after SD paradigm (*P* < 0.01 in the FST, *P* < 0.001 in the TST) compared with BP group, meaning SD treatment could induce depressive symptoms. However, CGA intervention reversed the phenomenon (*P* < 0.05 in the FST and TST) relative to SD group.

### 3.2. Biochemical Index Determination

**Figure 2:**
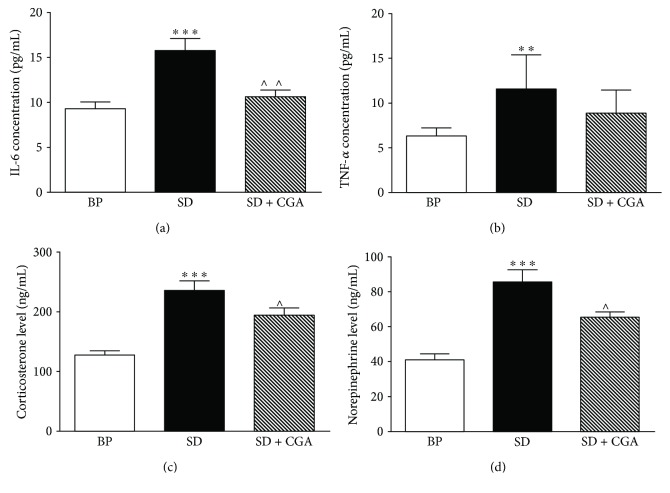
Biochemical index of the three rat group (BP: big platform group, SD: sleep deprivation group, SD + CGA: SD group with CGA intervention). Data are expressed as mean ± SEM (*n* = 6). Compared with the BP group, ^∗∗∗^*P* < 0.001; compared with the control group, ^∗∗^*P* < 0.01; compared with model group, ^∧∧^*P* < 0.01; and compared with SD group, ^∧^*P* < 0.05.

After SD treatment, the result of biochemical index (Figure 2) showed that the levels of serum IL-6 (*P* < 0.001), TNF-*α* (*P* < 0.01), CORT (*P* < 0.001), and NE (*P* < 0.001) were higher than those of control. However, after intervention of CGA for one week, these indexes were significantly reduced (*P* < 0.05) except for the level of TNF-*α*, which showed a tendency to decrease, yet.

### 3.3. Urine Metabolomics

#### 3.3.1. Multivariate Statistical Analysis of the Urine Metabolic Data

**Figure 3:**
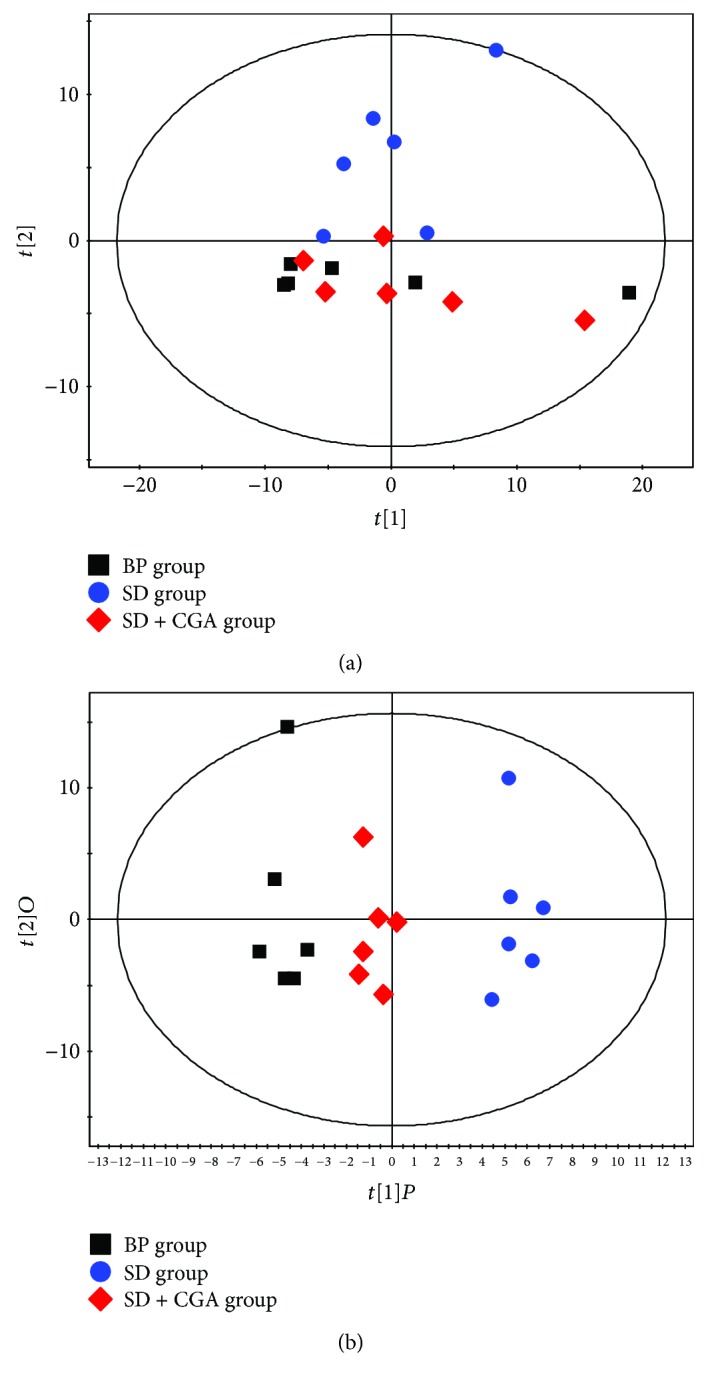
PCA and OPLS-DA score plot derived from the GC-MS analysis of urine from BP, SD, and SD + CGA groups.

PCA and OPLS-DA pattern were built to observe general clustering and trend among groups. Score plot analysis including Hotelling's *T*^2^ plot revealed no outliers. In the PCA score plot, the SD group was separated from the BP and SD + CGA group (R2X = 0.776, *Q*^2^ = 0.426) with little overlap, but no discernible clustering was observed between the BP and SD + CGA group (Figure 3(a)). However, in the OPLS-DA score plot, a clear separation was seen among three groups (Figure 3(b)). We found that SD samples were obviously discriminated from the BP group, and SD + CGA group showed a tendency to approach BP group (R2X = 0.758, R2Y = 0.975, *Q*^2^ = 0.853). The result indicated that the model was constructed successfully and it seemed that CGA treatment could improve urine metabolic alternation induced by SD model.

**Figure 4:**
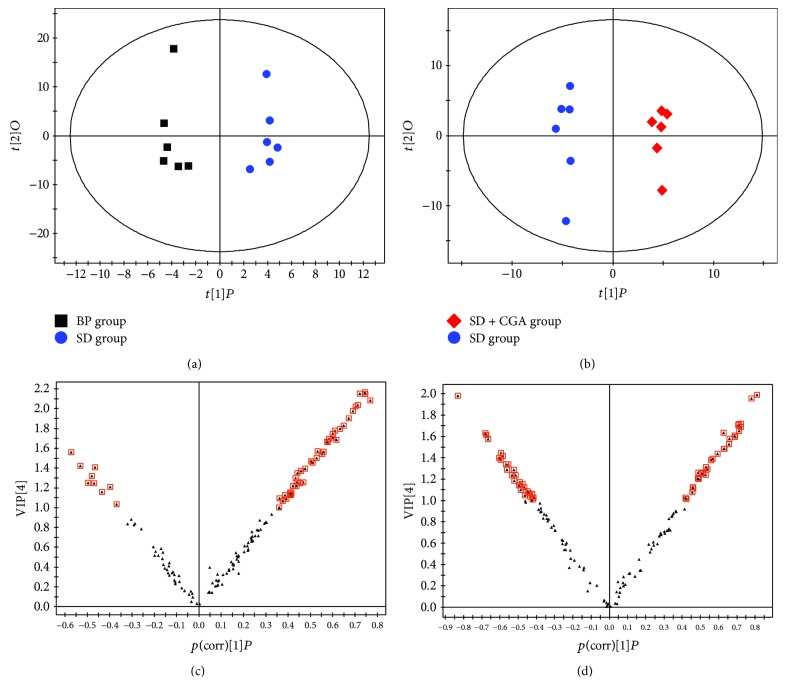
OPLS-DA score plots and V-plots between pairwise groups.

The OPLS-DA pattern was also applied to for pair-wise comparison and differential metabolites recognition. The BP and SD groups displayed significant deviation (R2X = 0.811, R2Y = 0.967, *Q*^2^ = 0.718) in the OPLS-DA plot (Figure 4(a)). OPLS-DA score plots (Figure 4(b)) also showed that the CGA-treated groups had distinctive metabolic profiles from the SD group (R2X = 0.776, R2Y = 0.988, *Q*^2^ = 0.797). It is worth noting that main automatic modeling parameters *R*^2^ and *Q*^2^ in pairwise groups were larger than 0.5, implying that patterns were robust and had good fitness as well as predictive ability.

#### 3.3.2. Identification of Differential Metabolites for SD and Interventional Effect of CGA

On the base of OPLS-DA analysis, the V-plot (Figures 4(c) and 4(d)) and variable importance for projection (VIP) statistics were used for selecting discriminating ions responsible for group separation [23, 24]. Ions were firstly selected with a VIP value threshold set to 1.0. Then, a two-tailed Student's *t*-test was carried out to further validate discriminating variables with a *P* value less than 0.05, for the purpose of decreasing false-positive ions. These discriminating metabolites were identified and listed in Table 2.

**Figure 5:**
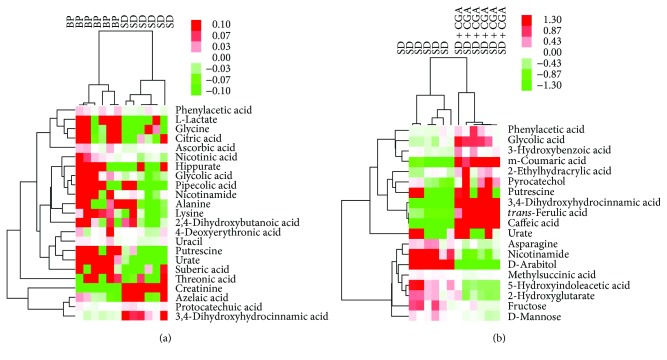
Heat maps described metabolite difference between pairwise groups.

Compared with BP group, 23 metabolites were significantly altered in SD group: creatinine, azelaic acid, protocatechuic acid, and 3,4-dihydroxyhydrocinnamic acid were upregulated, yet lactate, glycolic acid, alanine, nicotinic acid, phenylacetic acid, glycine, 4-deoxyerythronic acid, pipecolic acid, 2,4-dihydroxybutanoic acid, nicotinamide, uracil, threonic acid, suberic acid, putrescine, citric acid, hippurate, lysine, urate, and ascorbic acid were downregulated in SD group as shown in the heat map (Figure 5(a)). Intervention of SD rats with CGA moderated some metabolic alternations concomitantly with new metabolic changes as exhibited in the heat map (Figure 5(b)). Compared with SD rats, CGA raised glycolic acid, phenylacetic acid, putrescine, and urate apart from CGA conversion products (*trans*-ferulic acid, m-coumaric acid, 3,4-dihydroxyhydrocinnamic acid, and caffeic acid) [25]. In addition, CGA intervention solely increased 2-ethylhydracrylic acid, pyrocatechol, and 3-hydroxybenzoic acid, accompanied by decreased 4-deoxyerythronic acid, nicotinamide, asparagine, D-arabitol, fructose, D-mannose, 5-hydroxyindoleacetic acid, and 2-hydroxyglutarate. CGA modulated the above metabolites to display an antidepressant effect. These variations indicated that CGA not only exerted an antidepressant effect through modulating metabolic changes in SD rats but may also upregulate certain related compounds for a synergistic action.

#### 3.3.3. Metabolic Pathway Analysis for SD and Interventional Effect of CGA

**Figure 6:**
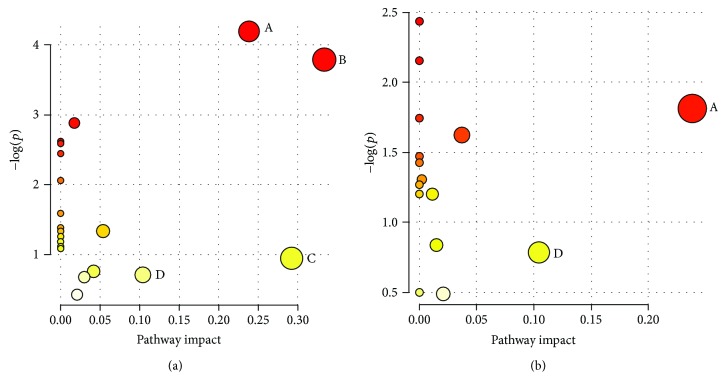
Summary of pathway analysis. (A) Nicotinate and nicotinamide metabolism, (B) glyoxylate and dicarboxylate metabolism, (C) glycine, serine, and threonine metabolism, and (D) arginine and proline metabolism.

In order to further explore the underlying mechanism about perturbed metabolites induced by SD model and interventional effect of CGA, a comprehensive metabolic network was mapped by means of MetaboAnalyst 3.0 (http://www.metaboanalyst.ca/) through integration of all potential biomarkers identified in present research [26]. As a result, four disturbed metabolic pathways were found to be the most relevant pathways involved in SD-induced metabolic dysbiosis (impact > 0.1) [27]. They were nicotinate and nicotinamide metabolism; glyoxylate and dicarboxylate metabolism; glycine, serine, and threonine metabolism; and arginine and proline metabolism (Figure 6(a)). After CGA intervention, we observed that glyoxylate and dicarboxylate metabolism and glycine, serine, and threonine metabolism had little impact value, suggesting that CGA may play an intervening role through these two pathways (Figure 6(b)). The remaining two pathways which did not change could be potential targets for future drug design.

## 4. Discussion

### 4.1. CGA Intervention Reversed Depression-Like Behavior and Biochemical Alternation Induced by SD

In many studies related to SD, a close correlation between SD and depression has been established. Some researches demonstrated a positive connection, saying that SD could weaken depressive symptoms [28, 29], while others are on the opposite [30, 31]. Here, we applied the FST and TST to evaluate depressive symptoms of the rats. The results strengthened the point that SD could lead to a depression-like phenotype. And that CGA really could exert an antidepressant effect as mentioned earlier [12]. A great deal of research has shown that proinflammatory cytokines, such as IL-6, are possibly contributed to the emergence of depression-like symptoms [32] and sleep perturbations [33, 34]. After 7 days of experiment, the accumulation of proinflammatory cytokines (IL-6, TNF-*α*) was consistent with previous research [35], which validated the point that disturbance of the circadian clock by SD was involved in the regulation of inflammation [36]. As a consequence of SD intervention, changes in the concentration of the stress response hormones (CORT and NE) were observed in accord with published paper [37]. Serum CORT is a vital central nervous excitation enhancer, which is responsible for stress in rat by modulating hypothalamic-pituitary-adrenocortical (HPA) axis. Here, growing serum CORT was regarded as a biological indicator of SD stress [38]. Based on highly expressed brain adrenergic receptor mRNA levels, research demonstrated that norepinephrine system partly participated in chronic sleep restriction [39]. Therefore, higher levels of serum NE in SD group may confirm a possible neurochemical mechanism underlying SD. It is gratifying that CGA intervention reversed almost all stress-induced biochemical changes. All results above proved that depression could not only be caused by SD but also be attenuated or reversed by CGA to some extent.

### 4.2. Biochemical Interpretation

By applying the rat model of SD and the GC-MS metabolomics coupled with multivariate statistical analysis, the urine metabolic characteristics of CON, SD, and CGA-intervened rats were described. Thirty-six differential metabolites were identified as displayed in Table 2. Most interestingly, some of the metabolites such as critic acid, azelaic acid, alanine, and glycine have been identified as biomarkers for depression [40]. Through pathway analysis, we found four pathways to be disturbed after SD paradigm. In particular, two pathways were reversed after CGA intervention.

#### 4.2.1. Disturbed Nicotinate and Nicotinamide Metabolism

In this altered pathway, nicotinic acid and nicotinamide were significantly reduced (*P* < 0.01) in SD group relative to BP group. Nicotinic acid, also known as vitamin B3, one of the thirteen essential vitamins for the human body, could be transformed into nicotinamide *in vivo*. As the major precursor of the coenzyme nicotinamide adenine dinucleotide (NADH/NAD^+^), nicotinamide is crucial to life. In cells, it participates in the synthesis of NAD^+^ and NADP^+^, which are coenzymes involved in a wide variety of enzymatic oxidation-reduction reactions for energy production, and glycolysis, tricarboxylic acid cycle (TCA cycle), and the electron transport chain are the most notable events of these [41]. Here, reduction of nicotinamide in the urinary metabolites may be owing to oversynthesis of NAD^+^ and NADP^+^. To a certain degree, the symptom implied energy metabolism imbalance in rat organism. In the meantime, intervention with CGA did not have a regulatory effect on nicotinate and nicotinamide metabolism perturbation. But it could be potential targets for future drug design.

#### 4.2.2. Disturbed Glyoxylate and Dicarboxylate Metabolism

The most significant alterations were attributed to changes in glyoxylate and dicarboxylate metabolism which include a variety of reactions involving glyoxylate or dicarboxylates. Glyoxylate is the conjugate base of glyoxylic acid. Likewise, dicarboxylates are the conjugate bases of dicarboxylic acids, a general class of organic compounds containing two carboxylic acid groups, such as oxalic acid or succinic acid. The glyoxylate cycle describes an important subset of these reactions involved in biosynthesis of carbohydrates from fatty acids or two-carbon precursors which enter the system as acetyl-coenzyme A. Our study found that two metabolites glycolic acid and citrate were involved in this metabolic process with marked reduction (*P* < 0.01) in SD rats. Interestingly, the alternation of glycolic acid was also found in the peripheral blood mononuclear cells from a rodent model of depression [42]. It is known to us all that citric acid plays a vital role in physiology which transforms fats, proteins, and sugars into carbon dioxide and provide the main energy for organisms. The alternations of metabolites strengthened the former point for energy metabolism disorder. The CGA in the current study obviously restored glyoxylate and dicarboxylate metabolism by upregulated glycolic acid.

#### 4.2.3. Disturbed Glycine, Serine, and Threonine Metabolism

The glycine, serine, and threonine metabolic pathway has been thought to provide a major energy metabolism precursor substance for TCA cycle [43]. Here, the core compound glycine in the pathway was decreased (*P* < 0.01) dramatically with a similar variation trend in the peripheral blood mononuclear cell of rat with chronic unpredictable mild stress rat model of depression [42], in the brain of depressive mice with chronic imipramine treatment [44] and in the serum of MDD patients [45]. Glycine is a simple, nonessential amino acid, which is involved in the production of phospholipids and collagen and releases energy, as well [46]. Literature documented that the concentration of plasma glycine may be responsible for regulating the mobilization of amino acids from peripheral tissues [47]. Glycine was also known as an inhibitory neurotransmitter, which possessed the ability to combine correlative NMDA receptor antagonist so as to exert an antidepressant effect [48]. Although after CGA treatment, the impact value of glycine, serine, and threonine metabolism decreased, we did not find any relative metabolites got modulated. The question was warranted to be solved in the future.

#### 4.2.4. Disturbed Arginine and Proline Metabolism

Arginine and proline metabolism is one of the central pathways for the biosynthesis of the amino acids. In addition to our discovery about disturbed arginine and proline metabolism after SD treatment, other researchers also found this significantly perturbed pathway in the prefrontal cortex of depressive rat model [24, 49]. We found that putrescine in the core position was the only disturbed metabolite (*P* < 0.01) in this pathway and had a consistent variation trend with published literature [50]. Actually, the small polyamine putrescine (1,4-diaminobutane) is ubiquitously and readily found in all three domains of life. It is a precursor, through N-aminopropylation or N-aminobutylation, to biosynthesize longer polyamines spermidine, sym-homospermidine, spermine, and thermospermine or even longer and branched chain polyamines, which enhance phosphorylation processes of functional proteins in neurons involved in the therapeutic mechanisms of antidepressants [51]. Putrescine is also biochemically modified for purposes of metabolic regulation and catabolism. Previous studies proved that polyamine levels are also possible to be regulated by stress and might play a role in depression. For instance, the levels of putrescine in the nucleus accumbens, spermidine, and spermine in the hippocampus were rescored after antidepressant treatment [50]. A research by administering spermidine intracerebroventricularly further revealed the antidepressant-like activity of the spermidine [52]. In light of existing studies, we hypothesized that the metabolic change of putrescine not only is too simple but also may be related to the development of depression; further research is warranted.

### 4.3. Metabolite-Energy Network

**Figure 7:**
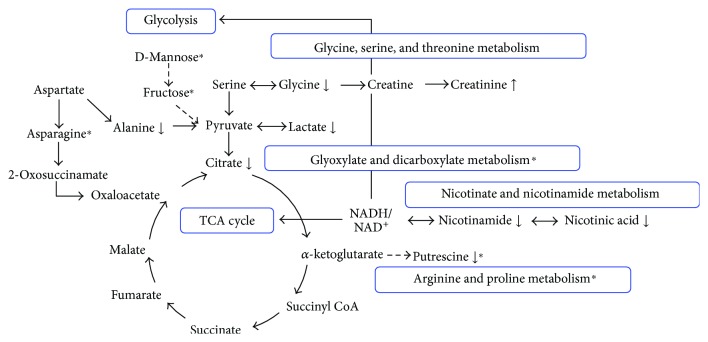
The energy perturbed process in response to SD exposure and CGA intervention. The levels of changed metabolites in SD group compared to BP group were labeled with (↓) downregulated or (↑) upregulated; ^∗^metabolites or metabolic pathway could be regulated by CGA. Solid arrows represent direct connections, and dashed arrows represent multiple and indirect connections between two compounds. The contents of the blue box represent energy processes or metabolic pathways.

Depending on metabolites discussed above and KEGG Pathway Database (http://www.genome.jp/kegg/), we drew a diagram concerning energy metabolism process (Figure 7). All in all, these findings corroborated previously published works which indicated that depression may be a kind of energy metabolic disorder.

Limitations to this study must be declared. First of all, the sample numbers in this study were relatively small; thus, future studies with larger cohorts should be performed to validate our findings. Regrettably, nearly all experimental rats were involved in stress. Actually, the CON group rats in normal cage-housing should be taken into consideration. In terms of value, our studies displayed how SD affected rats' organism alterations. Finally, owing to the diverse biochemical properties and wide concentration range of metabolites, further metabolomic methods or multiple metabolomic platforms (i.e., targeted metabolomics and nuclear magnetic resonance) should be employed in future studies.

## 5. Conclusion

It is the first time for us to demonstrate that SD indeed caused depressive symptoms in rat experiments. Depression-like behavioral phenotypes are reflected by alterations in serum inflammatory cytokines and hormone levels, and alternation of urinary metabolites. We further suggest that the development of depression is involved in alternations of energy-related pathways. CGA displays an antidepressant role by regulating inflammatory cytokines and hormone levels and metabolite related pathways. This research opens up new sights for a functional link between sleep disturbance and depression as well as the molecular mechanism of CGA in SD paradigm of depression.

## Figures and Tables

**Table 1:** Temperature program of column incubator in GC-MS.

Rate (°C·min^−1^)	Temperature (°C)	Hold time (min)
/	70	2
2.5	160	0
5	240	16

**Table 2:** Discriminating urinary metabolites of three groups.

RT (min)	Metabolite	SD versus BP		SD + CGA versus SD		
		Fold change^a^	*t*-test (*P*)	Fold change^a^	*t*-test (*P*)	
5.39	Lactate	0.42	0.02	—	—
5.62	Glycolic acid	0.55	0.00	1.68	0.01
6.01	Alanine	0.59	0.03	—	—
7.83	2-Ethylhydracrylic acid	—	—	3.21	0.00
8.87	Nicotinic acid	0.31	0.04	—	—
8.93	Phenylacetic acid	0.33	0.00	4.43	0.02
9.01	Glycine	0.58	0.01	—	—
9.19	Pyrocatechol	—	—	2.71	0.05
9.32	Methylsuccinic acid	—	—	0.57	0.01
9.56	4-Deoxyerythronic acid	0.36	0.05	—	—
9.98	Pipecolic acid	0.40	0.01	—	—
10.79	2,4-Dihydroxybutanoic acid	0.58	0.05	—	—
12.14	Nicotinamide	0.44	0.00	0.48	0.05
12.25	Uracil	0.62	0.01	—	—
12.56	Asparagine	—	—	0.34	0.00
13.66	Creatinine	2.28	0.01	—	—
13.79	Threonic acid	0.53	0.01	—	—
13.95	3-Hydroxybenzoic acid	—	—	3.14	0.00
14.32	Suberic acid	0.50	0.00	—	—
17.46	D-Arabitol	—	—	0.57	0.00
17.81	Putrescine	0.38	0.00	2.00	0.04
19.33	Azelaic acid	3.45	0.05	—	—
19.81	Protocatechuic acid	1.63	0.03	—	—
19.82	Citrate	0.42	0.00	—	—
20.28	Hippurate	0.45	0.04	—	—
20.97	Fructose	—	—	0.35	0.03
21.14	m-Coumaric acid	—	—	14.21	0.00
22.24	Lysine	0.46	0.03	—	—
22.73	3,4-Dihydroxyhydrocinnamic acid	9.48	0.02	87.95	0.02
26.08	*trans*-Ferulic acid	—	—	30.35	0.01
26.30	Urate	0.42	0.00	1.69	0.03
26.82	D-Mannose	—	—	0.51	0.03
26.96	Caffeic acid	—	—	59.50	0.01
27.43	5-Hydroxyindoleacetic acid	—	—	0.26	0.01
28.13	2-Hydroxyglutarate	—	—	0.43	0.05
33.58	Ascorbic acid	0.53	0.02	—	—

Note: ^a^fold change = SD (SD + CGA)/BP (SD); —: unable to be detected.

## Abbreviations

SD: Sleep deprivation
GC-MS: Gas chromatography coupled to mass spectrometry
CGA: Chlorogenic acid
MDD: Major depressive disorder
BP: Big platform group
SD + CGA: CGA-treated group
FST: Forced swimming test
TST: Tail suspension test
IL-6: Interleukin- 6
TNF-*α*: Tumor necrosis factor alpha
NE: Norepinephrine
CORT: Corticosterone
PCA: Principal component analysis
OPLS-DA: Orthogonal projections to latent structures discriminant analysis.
